# Cortical Neurotransmitters Measured by Magnetic Resonance Spectroscopy Change Following Traumatic Brachial Plexus Injury

**DOI:** 10.1055/a-2505-5657

**Published:** 2025-01-28

**Authors:** Ryckie G. Wade, Gráinne Bourke, Alexandra M. Olaru, Stephen R. Williams, David Shelley, Sven Plein, Robert D. Bains, James D. Bedford, Lucy E. Homer Newton, Chye Yew Ng, Laura Parkes, Caroline Lea-Carnall

**Affiliations:** 1 Department of Plastic and Reconstructive Surgery, Leeds Teaching Hospitals Trust, Leeds, United Kingdom; 2 Leeds Institute for Medical Research, University of Leeds, Leeds, United Kingdom; 3 Siemens Healthcare Ltd., Park View, Watchmoor Park, Camberley, Surrey, United Kingdom; 4 Division of Informatics, Imaging and Data Sciences, University of Manchester, Manchester, United Kingdom; 5 The Advanced Imaging Centre, University of Leeds, Leeds, United Kingdom; 6 Leeds Institute for Cardiovascular and Metabolic Medicine, University of Leeds, Leeds, United Kingdom; 7 Department of Plastic Surgery and Burns, Manchester University NHS Foundation Trust, United Kingdom; 8 Wrightington Wigan and Leigh NHS Foundation Trust, Wigan, United Kingdom; 9 School of Health Sciences, Manchester Academic Health Science Centre, Faculty of Biology, Medicine and Health, University of Manchester, Manchester, United Kingdom

**Keywords:** brachial plexus injury, spectroscopy, plasticity, motor, sensory, neurometabolites, GABA, NAA

## Abstract

**Introduction** GABA (γ-aminobutyric acid) is the major inhibitory neurotransmitter in the brain. In response to injury within the central nervous system, GABA promotes cortical plasticity and represents a potential pharmacological target to improve functional recovery. However, it is unclear how GABA changes in the brain after traumatic brachial plexus injuries (tBPIs) which represents the rationale for this pilot study.

**Methods** We serially scanned seven males (mean age 42 years [SD 19] without head injury) up to 19 months after tBPIs. T1-weighted images (1-mm isotropic resolution) and J-edited spectra (MEscher–GArwood Point RESolved Spectroscopy [MEGA-PRESS], TE 68 ms, TR 2,000 ms, 2 cm isotropic voxels) were acquired using a MAGNETOM Prisma 3T (Siemens Healthcare, Erlangen, Germany). Data were analyzed in jMRUI blind to clinical information to quantify GABA, creatine plus phosphocreatine (Cr), and N-acetylaspartate (NAA) concentrations. Additionally, gray matter and white matter proportions were assessed using SPECTRIM software. Interhemispheric means were compared using linear methods. Confidence intervals (CIs) were generated to the 95% level.

**Results** Within weeks of injury, the hemisphere representing the injured upper limb had a significantly lower GABA:NAA ratio (mean difference 0.23 [CI 0.06–0.40]) and GABA:Cr ratio (mean difference 0.75 [CI 0.24–1.25]) than the uninjured side. There were no interhemispheric differences in NAA:Cr. By 12 months post-injury, interhemispheric differences in metabolite concentrations equalized. There was no difference in the proportion of gray matter, white matter, or cerebrospinal fluid between the injured and uninjured hemispheres.

**Conclusion** After brachial plexus injuries, there are interhemispheric differences in GABA concentrations within the sensory and motor cortex. This represents a potential pharmacological target that warrants further investigation.

## Introduction

Cortical representations within the sensory and motor cortices have been shown to “remap” following learning 1 2 3 or after injury 4 over a time course of minutes to hours. The speed of cortical reorganization indicates that the mechanism underlying this phenomenon is likely to be via the “unmasking” of existing connections rather than synaptogenesis. Animal studies have demonstrated that a reduction in GABA (γ-aminobutyric acid), the brain's major inhibitory neurotransmitter, is crucial to this process. 5

Following injury to the brain (e.g., traumatic brain injury, 6 transient ischemic attack, 7 and stroke 8 ), GABA levels reduce in the sensory and motor cortices of the symptomatic hemisphere. Downregulation of GABA (reducing GABAergic inhibition) promotes plasticity analogous to long-term potentiation (LTP), allowing latent connections to strengthen and lost function to be recovered. In humans and animals, pharmacological modulation of GABA either prevents or facilitates LTP-like plasticity depending on the direction of modulation. 9 10 For example, reducing GABAergic tone has been shown to promote plasticity and functional remapping in the motor cortex after stroke. 11 Consequently, GABA modulation represents a potential pharmacological target that may improve central plasticity following injury and therefore, functional recovery.

**Fig. 1:**
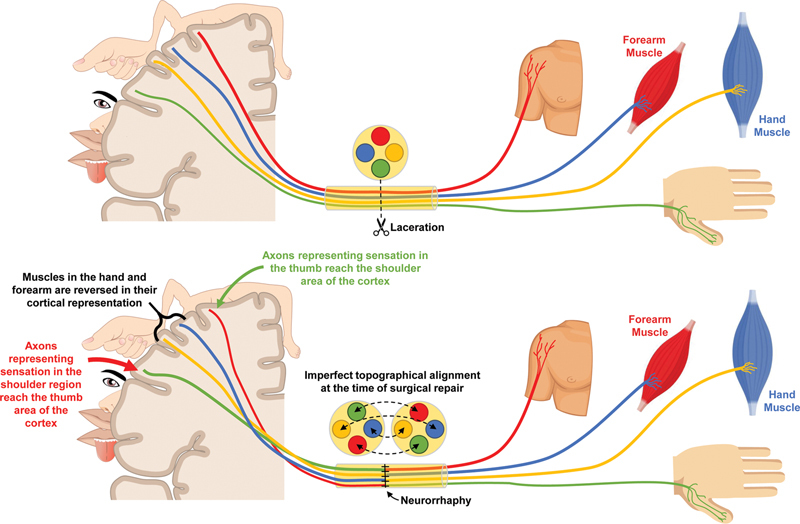
After division of a peripheral nerve (top) and surgical repair (bottom), the topography of the repair site is imperfect, so the cortical representation must change.

The brachial plexus is the network of nerves which innervate the upper limb. In England, there are approximately 20,000 cases of major trauma annually, 12 and 1% sustain traumatic injuries to their brachial plexus. 13 Traumatic brachial plexus injuries (tBPIs) are associated with disability, 14 15 chronic pain, 16 psychological morbidity, 17 and reduced quality of life. 14 15 These life-changing injuries have been estimated to directly cost health services in the United States $38,318 per patient 18 with an indirect cost of $1.1 million per patient. 19 Consequently, one in three patients with brachial plexus injuries (BPIs) are at risk of catastrophic health expenditure, whereby out-of-pocket health spending exceeds 40% of their post-subsistence income. 20 Surgical reconstruction of injured nerves is the mainstay of treatment; however, there are tens of thousands of axons in each nerve within the brachial plexus 21 and when surgically repaired, perfect topographical alignment is impossible. This means that regenerating motor neurons may synapse with different muscles and sensory neurons may innervate different cutaneous targets, necessitating cortical reorganization ( Fig. 1 ). Alleviation of GABA-driven synaptic inhibition is essential to facilitate learning and plasticity mechanisms, but no studies have explored GABA modulation after peripheral nerve injury.

Measurement of neurometabolites in vivo in the brain is possible using proton ( ^1^ H) magnetic resonance spectroscopy ( ^1^ H-MRS). The most studied neurometabolites include glutamate, N-acetylaspartate (NAA), creatine plus phosphocreatine (Cr), and choline-containing compounds (Cho). Although GABA is present in the spectra, the relatively low concentration (1–2 mM 22 ) and spectral overlap with other neurometabolites (e.g., creatine at 3 ppm) 23 24 means that J-difference editing (as implemented in MEscher–GArwood Point RESolved Spectroscopy, MEGA-PRESS) 25 is necessary to separate the GABA signal contaminated by other molecules.

We aimed to quantify the concentrations of GABA, Cr, and NAA in the brain and explore how they change in adults with acute BPI, using ^1^ H-MRS.

## Methods

This cohort study was designed and reported in accordance with the STROBE and STARD guidance, taking into account the domains of the QUADAS-2 26 and PRISMA-DTA 27 tools. Approval was provided by the United Kingdom National Health Research Authority (ID 19/NW/0324) and conducted in accordance with the relevant guidelines and regulations. Written informed consent was obtained from all participants. The raw MRS data are available open-source at https://osf.io/un24g/ .

### Recruitment

Between January 2019 and July 2020, we screened 22 consecutive adults with acute tBPI admitted to three Major Trauma Centers within England. Fifteen were excluded for the following reasons: four declined to participate or were uncontactable, four required immediate surgery which precluded preoperative MRI, three were unable to meet COVID-related isolation requirements, two were claustrophobic, one had learning difficulties and so could not provide informed consent, and one patient had a cervical spine injury requiring extended immobilization in a collar.

### Image Acquisition

Data were acquired at a field strength of 3 Tesla (T) using a MAGNETOM Prisma (Siemens Healthcare, Erlangen, Germany) with a 64-channel head coil. Firstly, we acquired a Magnetization Prepared RApid Gradient Echo (T1-weighted) dataset with the following parameters: TI 900 ms, TE 2.98 ms, TR 2,300 ms, flip angle 9 degrees, 176 sagittal slices, field of view 256 × 248, 1 mm isotropic resolution, GRAPPA factor 4, TrueForm B1 shim, no partial Fourier, AP phase encoding and bandwidth 240 Hz/Px. Thereafter, we acquired J-edited spectra using MEGA-PRESS 25 from 2-cm isotropic voxels centered on the right and left “hand knob” areas (using the “delta sign” on axial slices), respectively, with the following parameters: TE 68 ms, TR 2,000 ms, 64 averages, water suppression bandwidth 50 Hz, automated field map-based B0 shim, TrueForm B1 shim, 4 preparation scans, edit pulse frequency 1.9 ppm, edit pulse bandwidth 50 Hz, center frequency 4.7 ppm, receiver bandwidth 1,200 Hz for an acquisition time of 853 ms. Spectra were interleaved every acquisition between “edit-on” and “edit-off.” Real-time frequency adjustment was performed automatically on the scanner to prevent field drift affecting the difference spectra. The total scan time was 4 minutes 24 seconds per hemisphere. Data were stored as averaged fids for the “edit-on” and “edit-off” subspectra and exported from the scanner as .RDA files.

### Spectroscopic Processing

After acquisition, a custom-made processing script was used to generate phase-corrected spectra for the “edit-on” and “edit-off” conditions as well as the edited (difference spectrum) itself. Anonymized data were imported into jMRUI 28 v5.2 ( http://www.mrui.uab.es/mrui/ ) for analysis by S.R.W. and C.L-C., who were blind to clinical information. Prior to analysis using AMARES, 29 residual water was removed from the spectra using the HLSVD routine. The edited spectra were fitted with the following prior knowledge: NAA: relative phase 180 degrees, lorentzian line shape, frequency, linewidth and amplitude unconstrained; GABA: relative phase 0 degree Gaussian line shape, frequency and amplitude unconstrained, linewidth constrained between 15 and 25 Hz. Co-edited glutamate + glutamine: fitted as two peaks of equal amplitude separated by 10.25 Hz, relative phase 0 degree, lorentzian line shape, each linewidth constrained to equal that of NAA, frequency unconstrained. The prior knowledge for the edited GABA signal takes into account the contribution from co-edited macromolecules, 24 hence a gaussian line shape was assumed with constraints on the linewidth to force the fit to be meaningful. GABA measured from non-macromolecule-suppressed spectra is often referred to as GABA ^+^ , but we do not use this extra abbreviation for simplicity.

**Fig. 2:**
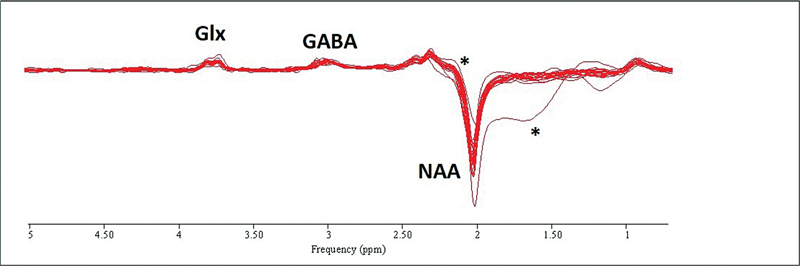
Overlay of 20 edited spectra. Peaks are assigned to NAA, GABA + macromolecules (GABA + ) and glutamate + glutamine (Glx). The two outlier spectra are indicated by *. The spectra have 6.0 Hz line-broadening applied for display purposes. GABA, γ-aminobutyric acid; NAA, N-acetylaspartate.

**Fig. 3:**
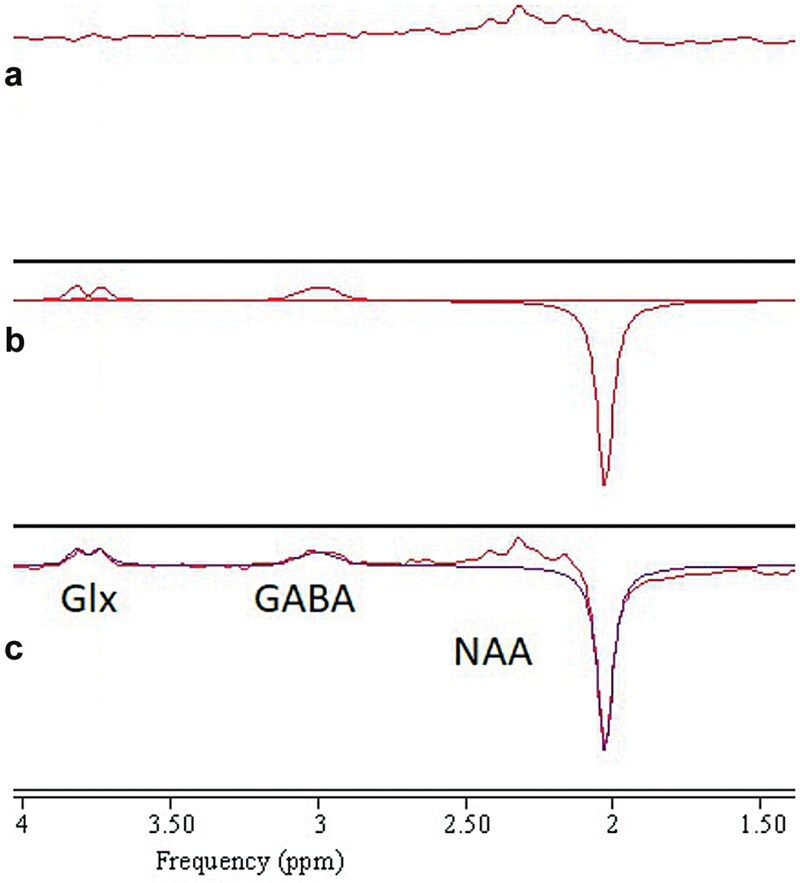
Example of AMARES fit to an edited spectrum. ( **a** ) residual, ( **b** ) individual fits, and ( **c** ) model fit overlaid onto original data with NAA, GABA + macromolecules and glutamate + glutamine (Glx) indicated. The spectra have had 6.0 Hz line-broadening applied for display purposes. GABA, γ-aminobutyric acid; NAA, N-acetylaspartate.

**Fig. 4:**
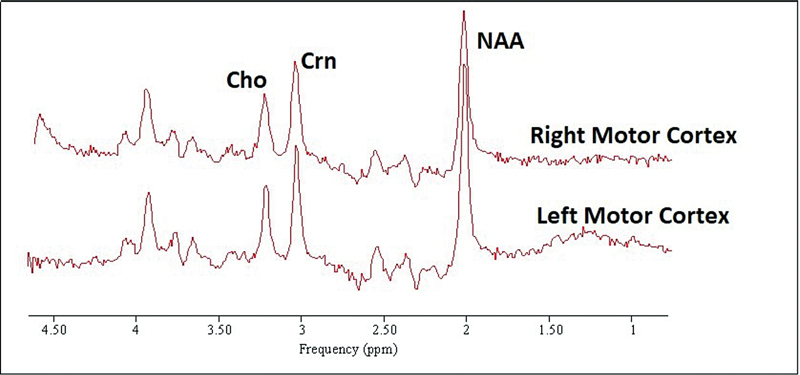
Example unedited (“edit-off”) spectra from the right and left hand knob areas of the motor cortex, with the quantified resonances indicated: NAA, N-acetylaspartate; Crn, creatine + phosphocreatine methyl; Cho, choline-containing compounds. The spectra have had 6.0 Hz line-broadening applied for display purposes. GABA, γ-aminobutyric acid.

The “edit-off” spectra were also quantified by AMARES with the following prior knowledge for NAA, Cr at 3.03 and 3.96 ppm and cho: amplitudes and frequencies were all unconstrained, lorentzian line shape was stipulated and linewidths were all constrained to equal that of Cr at 3.03 ppm. Example spectra, together with the AMARE fit for an edited spectrum are shown in Figs. 2 to 4 . To quantify GABA, Cr and NAA are assumed to be of quasi-constant concentration (both within and between subjects) such that GABA:Cr and GABA:NAA ratios may be computed as a proxy for GABA concentration. The edited signal for NAA was used to construct the GABA ratio, since any slight missetting or drift of the amplitude or phase of the pulse would affect both the edited GABA and NAA signals similarly.

The quality of the spectra was assessed by visual inspection and by measuring the linewidth and Cramer–Rao lower bound (CRLB) of the AMARES 29 fit for GABA in the edited spectra. The signal-to-noise ratio of NAA was also recorded for edited spectra.

Additionally, gray matter and white matter proportions were assessed using SPECTRIM. 30 This provides a graphical user interface to enable segmentation of the T1-weighted anatomical DICOM images, with extraction of the gray matter, white matter, and cerebrospinal fluid (CSF) content of the spectroscopic voxels. This is a necessary step to interpret any changes in GABA, as it is known that the GABA content of gray matter is approximately double that of white matter. 31

### Functional Outcomes Assessment

Grip strength was assessed using a digital grip dynamometer. Key (lateral) and tip (index pulp to thumb pulp) pinch were assessed using the Jamar Plus Digital Pinch Dynamometer For all assessments, three measurements were taken the best (strongest) was used.

### Analysis

Data were analyzed using Stata v16/MP (StataCorp LLC, TX). Scaled variables approximating the normal distribution are represented by the mean (and standard deviation, SD). To estimate the interhemispheric differences in the concentrations of neurotransmitters, means were compared by linear regression. As the dataset is small, we used resampling with replacement (bootstrapping) with 1,000 iterations to improve estimates of the variance. Age may affect GABA levels 32 33 but as there is no conceptual association between these variables and interhemispheric differences after nerve injury, adjustment was not required. Although the patients in this series were exposed to analgesics that modulate GABA and Glutamate (Gabapentin or Pregabalin), as we were directly comparing interhemispheric differences, the effect of these drugs will be neutralized, and so further adjustment is unnecessary. Confidence intervals (CIs) were generated to the 95% level.

## Results

Ultimately, seven males were included with a mean age of 42 years (SD 19, range 21–76). No patients had a head injury. Given the severity of the injuries sustained by patients, the nationwide COVID-19 measures in place during the study, and the need to accommodate other surgeries, patients were scanned at different time points ( Table 1 ) and there was variable attrition.

**Table 1:** The study timeline showing the months from injury to MRI (M) and/or surgery (S) for each patient, ordered by age

Patient	Months from injury																			Mechanism of injury	Injury pattern	Surgery
	1	2	3	4	5	6	7	8	9	10	11	12	13	14	15	16	17	18	19			
21♂	M																			Mountain bicycle crash	Upper trunk and axillary nerve	
28♂	M																			Rugby injury	Upper trunk	
35♂			M																	Motorcycle RTC	Upper trunk and posterior cord	
31♂																			M	Pedestrian versus car	Post-ganglionic C5-7	
47♂	M	S					M								M					Motorcycle RTC	Post-ganglionic C5-6	Neurolysis
51♂						M									M					Motorcycle RTC	Post-ganglionic pan-plexus injury	
76♂			S				M													Traction injury	Upper and middle trunks	Neurolysis

Abbreviation: RTC, road traffic collision; S, surgery; M, MRI.

Details of the injury and surgical reconstruction are shown also.

### Patient Characteristics

Five adults were involved in vehicular collisions, one sustained a rugby “stinger” injury, and one sustained a traction injury from horse reigns. At the time of scanning, patients were consuming daily a mean of 1 g of paracetamol, 171 mg of NSAIDs, 24 mg of opioids (in morphine-equivalent units), and 21 mg of pregabalin. None received benzodiazepines or other neuromodulating agents.

Using the EQ-5D-5L, patients reported a median overall quality of life of 85% (interquartile range [IQR] 70–89). However, the summary scores were considerably lower for upper limb-specific tools, such as the impact of hand nerve disorders (I-HaND) (27%, IQR 11–46), Quick-disability of the arm, shoulder and hand (DASH) (42%, IQR 21–75), and brachial plexus assessment tool (BRaT) (62%, 7–84). The median visual analogue score for pain within the neck, arm, forearm, and hand were 20% (IQR 7.5–29), 9% (IQR 0–31), 21% (IQR 5–34), and 20% (IQR 4–36), respectively. Patients had worse motor deficits than sensory deficits, as shown in Table 2 whereby strength in the injured limb was approximately half that of the normal limb, while the objective sensory tests, including the static two-point discrimination (median 2, IQR 2–4) and Semmes–Weinstein Monofilament (median 0.07, IQR 0.07–4) were within the normal range.

**Table 2:** Patient characteristics at the time of their first scan

	Uninjured limb	Injured limb	Mean difference (95% CI)
Mean grip strength in kg (SD)	44 (31–51)	22 (19–42)	18 (6, 31)
Mean key pinch in kg (SD)	8.4 (1.8)	6.0 (2.8)	2.0 (−1.1, 2.05)
Mean index-to-thumb pinch in kg (SD)	5.0 (2.50)	3.6 (1.87)	1.3 (−0.5, 3.1)

Abbreviation: CI, confidence interval; SD, standard deviation.

### Magnetic Resonance Spectroscopy

Twenty spectra were collected from the seven subjects. Figure 2 shows all 20 edited spectra aligned and overlaid. The spectra are reproducible and of very similar quality except for two traces which show increased (more negative) and decreased NAA signals. The statistical analyses were run both with and without these two spectra and no meaningful differences were noted with a sensitivity analysis excluding the two outlier datasets, so a complete case analysis is presented. Quality assurance metrics (NAA and edited GABA linewidth, CRLB, and signal-to-noise ratio, Supplementary Table S1 [available in the online version only]) showing tight distributions of the parameters, consistent with the visual appearance of the overlaid spectra ( Figs. 2 3 4 ). Both GABA linewidth and CRLB remain within three median absolute deviations of the median value, while NAA linewidth lies outside this range in two spectra. In these spectra, the linewidth is still well within expected values for the human brain at 3 T, so this does not justify the exclusion of the data. An example of the AMARES fit to an edited spectrum is shown in Figs. 3 and 4 shows an example of an unedited (“edit-off”) spectra.

**Fig. 5:**
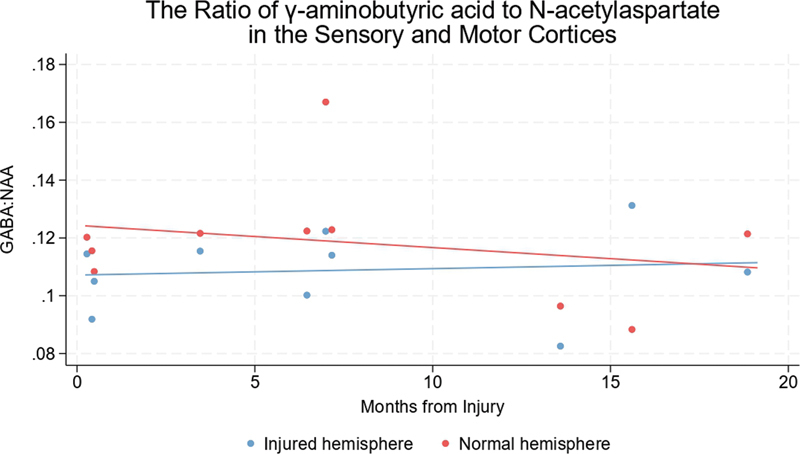
GABA:NAA ratios in the hemispheres representing the injured and uninjured limbs of adults with traumatic brachial plexus injuries. The linear fit indicates that GABA:NAA ratios fall in the hemisphere representing the injured limb (relative to the normal hemisphere) immediately after injury and normalize over months. GABA, γ-aminobutyric acid; NAA, N-acetylaspartate.

Those who had MRS within 3 months of their injury, the hemisphere representing the injured upper limb had a significantly lower GABA:NAA ratio as compared to the unaffected hemisphere (mean difference 0.23 [CI 0.06–0.40]). After approximately 6 months, the GABA:NAA difference had reduced to a mean of 0.14 (CI 0.03–0.25). Beyond a year, there was no detectable interhemispheric difference in GABA:NAA ( Fig. 5 ).

**Fig. 6:**
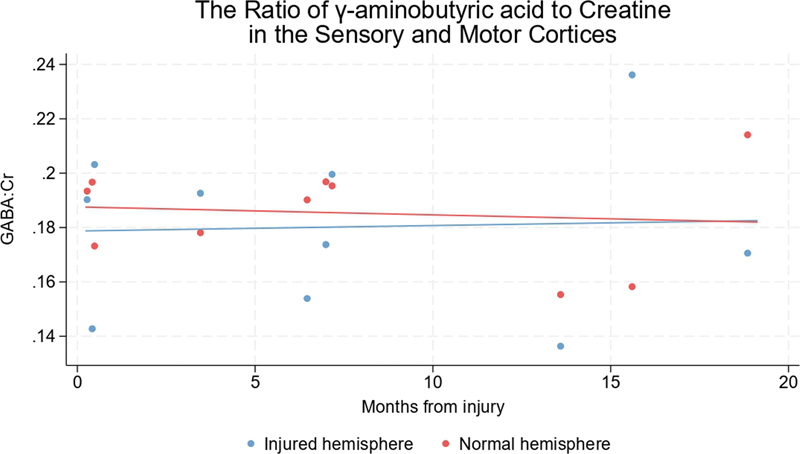
GABA:Cr ratios in the hemispheres representing the injured and uninjured limbs of adults with traumatic brachial plexus injuries. The linear fit indicates that GABA:Cr ratios fall in the hemisphere representing the injured limb (relative to the normal hemisphere) immediately after injury and normalize over months. Cr, creatine + phosphocreatine; GABA, γ-aminobutyric acid.

A similar pattern was observed for GABA:Cr ratio, whereby patients who were scanned early (within 3 months of their injury) had significantly lower GABA:Cr ratios in the hemisphere representing the injured limb (mean difference 0.75 [CI 0.24–1.25]). At approximately 6 months, the interhemispheric difference in GABA:Cr had reduced (mean difference 0.26 [CI −0.14, 0.66]) and by 1 year, it had equalized ( Fig. 6 ).

**Fig. 7:**
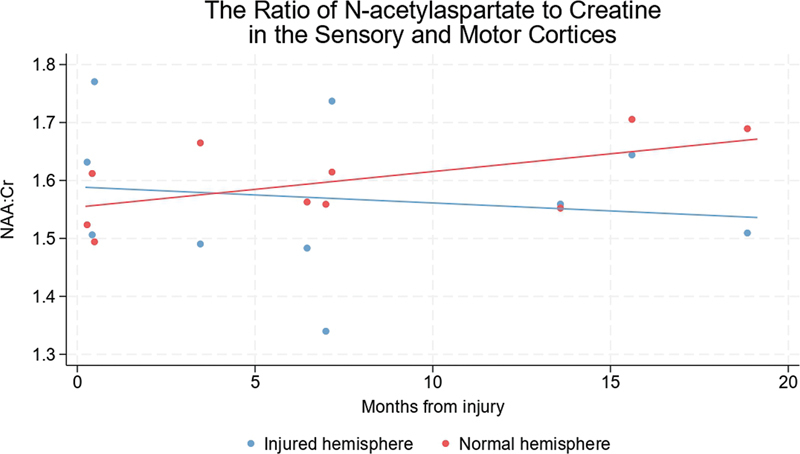
Change in NAA:Cr ratio in the hemispheres representing the injured and uninjured limbs of adults with traumatic brachial plexus injuries. The linear fit of the injured and normal hemispheres was not different. Cr, creatine + phosphocreatine; NAA, N-acetylaspartate.

**Fig. 8:**
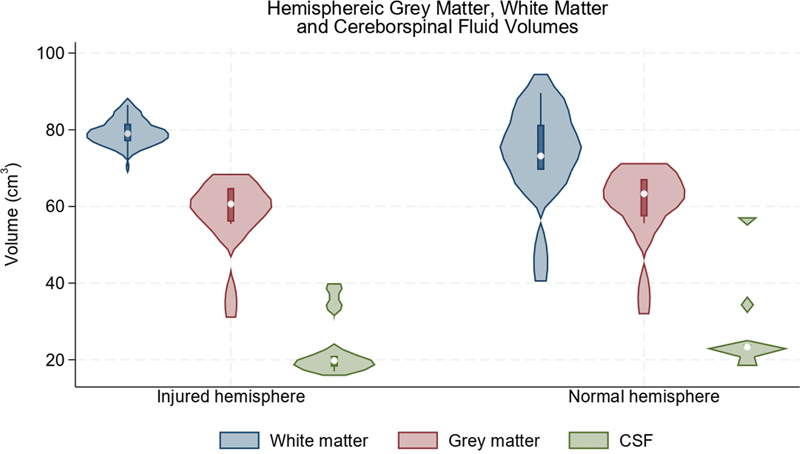
The averaged volumes of the gray matter, white matter, and cerebrospinal fluid (CSF) in the hemispheres representing the injured and uninjured limbs.

In contrast, we detected no significant interhemispheric differences in NAA:Cr at baseline or over time ( Fig. 7 ). Equally, there were no differences in the proportion of gray matter, white matter, and CSF between the injured and uninjured hemispheres ( Fig. 8 ). There were no appreciable linear associations between any neurotransmitter ratios and the I-HaND, EQ-5D-5L, Quick-DASH, or BrAT scores.

## Discussion

We show prolonged reductions in GABA concentrations within the sensorimotor cortex representing the injured limb, in patients with acute tBPIs. This window of GABA reduction (facilitating improved plasticity and cortical remapping) appears to last for several months but not beyond 1 year, meaning that surgeons should aim for their reconstructive procedures to be completed as soon as possible, in the knowledge that reinnervation of target organs make take months and the plastic potential of the brain declines over time.

Recent work has shown that following traumatic brain injury, 6 transient ischemic attack, 7 stroke, 8 and ischemic deafferentation of the upper limb, 34 GABA concentrations fall in the symptomatic sensory and motor cortices. Levy et al (2002) 34 showed that GABA concentrations fall within minutes of ischemic deafferentation of human limbs. The understanding of temporal changes in GABA was extended by Yasen et al (2018) 6 in their study of mild traumatic brain injury, whereby cortical GABA concentrations were low at 3 days post-injury (compared to controls) and remained low 2 weeks later. Similar patterns were seen in patients with an established stroke (mean 7 months old) whereby patients had lower cortical GABA levels than controls. 8 In the longer term, Tremblay and colleagues (2014) 35 showed that (independent of cortical atrophy) GABA levels normalize 3 years after brain injury. These studies agree with our observations in tBPI, whereby GABA concentrations fall rapidly after injury, remain low months later and normalize approximately 1-year post-injury. It is important to clarify the temporal changes in GABAergic inhibition (whether active or incidental) because GABA levels are known to modulate plasticity. Lower levels of GABA have been shown to promote plasticity and improve perceptual learning. 36 By reducing GABAergic inhibition, vacant neuronal connections are “unmasked” which enables them to form new synapses within minutes-to-hours, such that function can be recovered or new connections fortified. Given the severity of BPI, its wider impact on both patients and their families, and the extraordinary costs to the health services, the potential to pharmacologically modulate GABA (to maintain and extend the window of “improved central plasticity” for surgical reconstruction) might benefit patients and surgeons alike.

In both animals 37 38 and humans, 39 early nerve repair after BPI yields better outcomes. Recent work has shown that the time from injury to reconstruction in adults with BPI is linearly associated with functional outcomes, whereby each month of delay to neurotization reduces the odds of meaningful recovery by 7%. 39 Moreover, the choice of donor nerve(s) used for reconstruction directly influences the chance of useful recovery. 39 This observation cannot be completely explained by local anatomical factors (such as donor–recipient axon counts) and we speculate that the plastic potential of the cortex for some donor nerves may be greater than others. For example, the most prevalent donor nerves for restoring elbow flexion via neurotization of the musculocutaneous nerve are the Oberlin transfers 40 41 (utilizing fascicles from the median and/or ulnar nerves that natively perform wrist flexion) or intercostal nerve transfer (using nerves that provide somatic control of breathing). Although the median/ulnar fascicles (with a mean of 1,318 and 1,860 axons 21 ) and several intercostal nerves (with axon counts of 520–1,353 per nerve, depending on the level 42 ) are well-matched for size and axon counts to the musculocutaneous nerve (which has 1,600 efferent axons producing elbow flexion 21 ). there are systematic differences in the outcomes between these two transfers which implies that other factors are at play. In this paradigm, we hypothesize that the cortical areas controlling wrist flexion may be more amenable to adaption to controlling elbow flexion (after nerve transfer) than cortical areas designated for voluntary control of respiration. Whatever the mechanisms governing this change in cortical representation, the potential to modulate the plastic potential through GABA represents an important avenue for future research.

We were required to exclude some patients with more severe BPI or concurrent injuries. Equally, this population is difficult to access for research for many reasons: at best, patients are young, working-age adults with dependents and so have limited time to engage in research; at worst, they are polytraumatized, disabled, 14 15 suffering from chronic pain, 16 and psychopathology 17 and so cannot engage. Consequently, our data collection time points were inconsistent which could introduce time-related biases and the sample we ultimately recruited may not reflect the population. It is widely known that GABA:NAA ratios are inhomogeneous in the brain 43 so our findings may not be generalizable to other regions. Within the sensorimotor cortex, the hemisphere responsible for the dominant hand exhibits higher GABA concentrations 44 so without controlling for this confound (due to insufficient data), our results may not be precise.

## Conclusion

Our pilot data suggest that there may be a fading window of time (lasting approximately 12 months) where the brain has plastic potential and thus, is receptive to cortical reorganization following peripheral nerve repair. Future research should explore changes in GABA in other peripheral nerve disorders and whether modulation of GABA improves outcomes for patients.
