# Supplementary material for: Cortical Neurotransmitters Measured by Magnetic Resonance Spectroscopy Change Following Traumatic Brachial Plexus Injury
Source: J Brachial Plex Peripher Nerve Inj. 2025 Jan 28;20(1):e16–25. doi: 10.1055/a-2505-5657 (PMC11774636; doi:10.1055/a-2505-5657)
Supplement: Supplementary file 1 — Supplementary Material [file 10-1055-a-2505-5657-s2400004.pdf]

Supplementary Table S1 Quality assurance metrics: linewidth and signal-to-noise ratio are presented for N-acetylaspartate; linewidth and % Cramer–Rao lower bounds are presented for γ-aminobutyric acid

	NAA LW	GABA LW	NAA SNR	NAA LW (edit-off)	GABA % CRLB
Mean	6.7	24.0	41.6	6.4	15.2%
Range	4.6–12.1	18.0–30.0	26.1–56.6	4.7–13.2	10.8–19.5%
CoV	27%	20%	17%	30%	16%
MAD-range	4.2–8.1	15.0–39.3	29.5–55.3	4.3–7.5	10.2–19.7%

Abbreviations: CRLB, Cramer–Rao Lower Bounds; GABA, γ-aminobutyric acid; LW, linewidth; MAD-range, range of the median absolute deviation; NAA, N-acetylaspartate; SNR, signal-to-noise ratio.
Mean values, range, and % coefficient of variation are shown as the well as the MAD-range. This metric runs from the (median $-$ 3× the median absolute deviation) to (median $+$ 3× the median absolute deviation) and is considered a more reliable metric for outlier detection than mean $\pm$ 3× standard deviation.

 © 2025. The Author(s).
